# Co-Ingestion Timing, Rather than Food Matrix or Dose, Determines α-Tocopherol Bioavailability from Intrinsically Labeled Spinach in Healthy Adults: A Crossover Pharmacokinetic Study

**DOI:** 10.3390/antiox15091076

**Published:** 2026-08-28

**Authors:** Shahabeddin Rezaei, Sisi Cao, Min Zeng, Maria L. Antonius, Jillian T. Pierson, Ariana H. Bond, Allyson Q. McHenry, Joshua J. Blakeslee, Richard S. Bruno

**Affiliations:** 1 Human Nutrition Program, The Ohio State University, Columbus, OH 43210, USA; rezaei.17@osu.edu (S.R.); pierson.150@buckeyemail.osu.edu (J.T.P.);; 2 Department of Horticulture and Crop Science, The Ohio State University, Columbus, OH 43210, USA

**Keywords:** vitamin E, antioxidant, green leafy vegetables, egg, bioavailability, pharmacokinetics

## Abstract

Dark green leafy vegetables, such as spinach, are rich sources of α-tocopherol (α-T), a lipid-soluble antioxidant, but may contribute little to α-T status due to low intake and limited bioavailability. This study investigated whether egg co-consumption enhances α-T bioavailability from spinach and whether effects differ by egg dose, food matrix, or timing of intake. In a crossover study, healthy adults consumed deuterium-labeled (d_x_)-spinach (containing 5 mg d_x_-α-T) alone; or with 1, 2, or 3 hard-boiled eggs; 2 egg whites; or vegetable oil (9.6 g). Two exploratory trials evaluated delayed egg consumption 3 h after spinach intake. Compared with spinach alone, d_x_-α-T AUC_0–72h_ (µmol/L × h) was 1.5–1.8-times higher when spinach was co-consumed with 1, 2, and 3 eggs (*p* ≤ 0.05), respectively, with no egg dose-dependent differences (*p* > 0.05). Co-ingestion of spinach with egg whites or vegetable oil increased d_x_-α-T AUC_0–72h_ similar to whole eggs (*p* > 0.05). Delayed egg intake did not affect d_x_-α-T AUC_0–72h_ relative to spinach alone (*p* > 0.05). Plant-derived α-T bioavailability can be improved through co-ingestion with other foods but is independent of egg dose and food matrix. Effective absorption requires concurrent intake, indicating that timing and associated digestive processes are key determinants and support food-pairing strategies to improve vitamin E bioavailability and its antioxidant contribution to plant-based diets. Registered at ClinicalTrials.gov (NCT04287816).

## 1. Introduction

Vitamin E comprises eight structurally related tocopherols and tocotrienols, classified as α-, β-, γ-, and δ- forms [1]. Although all forms exhibit antioxidant function, only α-tocopherol (α-T) is considered essential in humans due to its ability to reverse clinical deficiency [2]. As a lipid-soluble antioxidant, α-T protects polyunsaturated fatty acids within cellular membranes by terminating lipid peroxidation chain reactions [3]. While overt α-T deficiency is rare [2], α-T inadequacy is prevalent. In the U.S., 92–98% of adults fail to meet the recommended intake of 15 mg/day [4] and ~65% have plasma α-T ≤ 30 µmol/L [5]. Current recommendations define α-T adequacy in plasma at ≥12 µmol/L based on erythrocyte resistance to hydrogen peroxide-induced hemolysis [2]. However, higher plasma concentrations (≥30 µmol/L) have been consistently associated with lower mortality and reduced risk of oxidative stress-related diseases, including cardiovascular disease and certain cancers [5,6,7,8,9]. Together, these findings indicate a gap between α-T adequacy and optimal health outcomes, emphasizing a critical need for strategies that improve α-T bioavailability from foods consumed within healthy dietary patterns. Given its central role as a chain-breaking antioxidant that limits lipid peroxidation, improving α-T bioavailability may enhance protection against oxidative stress-driven chronic diseases.

In the U.S., α-T intake is derived largely from foods that provide modest amounts but are consumed frequently [4]. As public health guidance increasingly emphasizes plant-rich diets [10], dark green leafy vegetables such as spinach have been highlighted as nutrient-dense sources of α-T [11]. However, their contribution to α-T status is limited due to low consumption, with ~80% of Americans failing to meet recommended intake levels for leafy greens [12], and poor α-T bioavailability [13,14]. The limited bioavailability reflects plant matrix characteristics, including rigid cell walls that limit nutrient bioaccessibility [15], and the inherently low lipid content of these vegetables, which is insufficient to support micellarization and intestinal absorption of fat-soluble vitamins [16].

α-T absorption occurs in the small intestine following incorporation into mixed micelles, a process dependent on fatty acids, bile salts, and pancreatic enzymes [1]. Although fat is recognized to enhance fat-soluble vitamin bioavailability [17], evidence specific to α-T is inconsistent [18,19,20]. For example, α-T bioavailability increases when α-tocopheryl acetate was ingested with fat [18], whereas ingestion of non-esterified α-T with dairy products varying in fat shows no enhancement [19,20]. Extrapolating these findings to plant-derived α-T is problematic. Plants contain only non-esterified α-T, whereas many studies provide esterified α-T that requires enzymatic hydrolysis prior to absorption. α-T in plants is embedded within membranes that limit bioaccessibility [21]. Further, leafy vegetables contain negligible lipid [22], making them poorly suited to support intestinal micellarization and chylomicron assembly required for α-T absorption [23]. Food pairing represents a practical strategy to overcome plant-specific barriers and enhance the bioavailability of lipophilic antioxidants such as α-T, thereby supporting their role in protecting against oxidative stress. Eggs are commonly consumed with leafy greens and have a distinctive lipid matrix composed of ~66% triglycerides and 28% phospholipids [24]. Compared with plant oils, which are triglyceride-based and contain minimal phospholipids [25], eggs may provide a more favorable matrix for α-T absorption. In addition to stimulating bile secretion via fatty acid-induced cholecystokinin release [26], egg-derived phospholipids may facilitate emulsification, micelle formation, and chylomicron assembly [27,28,29]. Indeed, consumption of eggs with mixed salads increases postprandial α-T in the triglyceride-rich fraction [30]. However, because eggs and vegetables contain α-T, and isotopic tracers were not used, the dietary origin of absorbed α-T could not be determined.

A critical gap remains regarding the extent to which dietary fat source, food matrix, and meal timing influence the bioavailability of plant-derived α-T. Moreover, α-T bioavailability from spinach has not been quantified in humans using intrinsic stable isotope labeling. Our study addressed this gap by examining egg co-ingestion on α-T bioavailability from intrinsically labeled spinach. We hypothesized that α-T bioavailability from spinach would increase in an egg-dependent manner, that phospholipid-rich eggs would enhance bioavailability to a greater extent than triglyceride-rich vegetable oil, and that delayed egg consumption would attenuate absorption. Using deuterium-labeled (d_x_) spinach in a controlled crossover pharmacokinetic study in healthy adults, this work provides evidence for food-based strategies to enhance α-T bioavailability and inform dietary guidance aimed at improving vitamin E status and optimizing antioxidant protection within plant-forward dietary patterns.

## 2. Materials and Methods

### 2.1. Study Design

**Figure 1:**
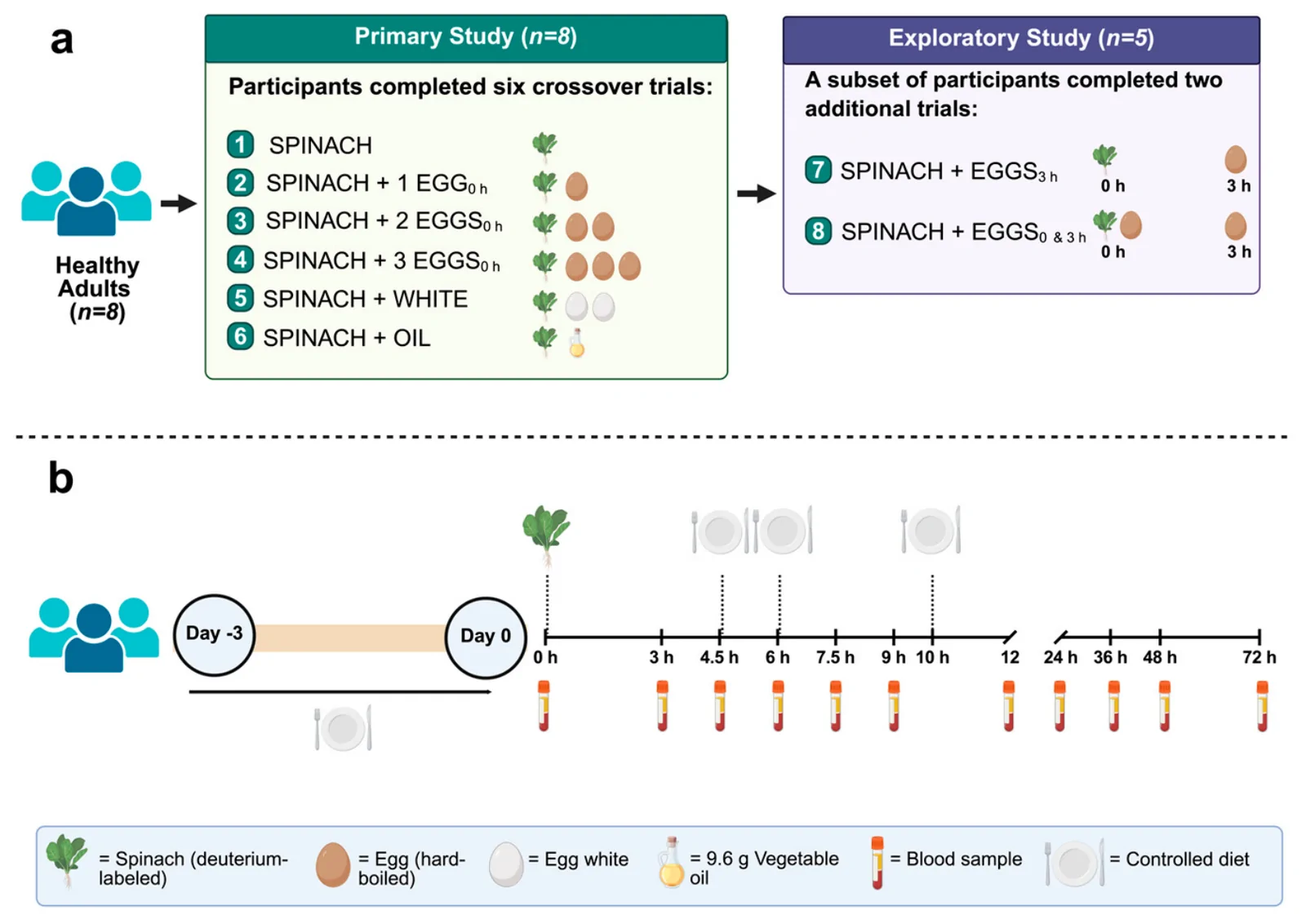
Study design and experimental timeline for evaluating dose, food matrix, and meal timing effects on spinach-derived deuterium-labeled-α-tocopherol (d_x_-α-T) bioavailability. (**a**) Study design and test conditions. Healthy adults participated in a controlled crossover study to evaluate egg dose, food matrix, and meal-timing effects on d_x_-α-T bioavailability. In the primary study (*n* = 8), participants completed six crossover trials: SPINACH, SPINACH + 1 EGG_0h_, SPINACH + 2 EGGS_0h_, SPINACH + 3 EGGS_0h_, SPINACH + WHITE, and SPINACH + OIL. A subset of participants (*n* = 5) completed two additional exploratory meal-timing trials: SPINACH + EGG_3h_ and SPINACH + EGGS_0&3h_. In all trials, participants consumed intrinsically labeled spinach providing a standardized dose of 5 mg d_x_-α-T. Whole eggs (hard-boiled) were provided at varying doses or time points; WHITE consisted of egg whites from two eggs; and OIL consisted of 9.6 g vegetable oil to match the fat content of two whole eggs. (**b**) Experimental timeline and sample collection. Participants followed a controlled diet for 3 days prior to each trial and during the first 12 h post-intervention. Daily diets provided 53 ± 0.5% of energy from carbohydrate, 16 ± 0.5% from protein, 31 ± 0.4% from fat, and 7.2 ± 0.3 mg/day of α-T. On Day 0, after collection of a baseline blood sample (0 h), participants consumed d_x_-spinach alone or with the designated test meal. Standardized meals were provided during the first 12 h, including lunch and a snack immediately after the blood collections at 4.5 and 6 h, respectively, followed by dinner at 10 h. Whole blood was collected at baseline and at timed intervals over 72 h post-ingestion. Plasma was isolated and analyzed by LC-MS to determine d_x_-α-T concentrations for pharmacokinetic assessment. Created in BioRender. Rezaei, S. (2026) https://BioRender.com/oqw0jbd, accessed on 18 August 2026.

This study was conducted as a controlled crossover pharmacokinetic trial in healthy adults with both primary and exploratory objectives (Figure 1). The primary objective (*n* = 8) was to determine egg dose and food matrix effects on the bioavailability of spinach-derived d_x_-α-T, whereas the exploratory objective (*n* = 5) examined whether the timing of egg consumption relative to spinach intake influenced its bioavailability.

The study was originally designed as a 6-arm crossover trial, with each lasting 72 h, to evaluate egg-dependent and food matrix-dependent effects on α-T bioavailability from d_x_-spinach. The clinical trial was conducted between June 2021 and June 2023. However, after initiation of the clinical trial, additional resources became available to incorporate an exploratory objective focused on meal timing, resulting in the addition of two exploratory arms (Figure 1). Due to these logistical constraints, the study proceeded in a non-randomized manner, and primary and exploratory objectives were analyzed separately. To minimize potential bias associated with the non-randomized design, all test meals were consumed on-site within 10 min under investigator supervision; therefore, participant behavior was unlikely to influence the physiological outcomes. Participants consumed a standardized diet before each trial to control dietary α-T intake, and each trial was separated by a washout period to minimize carryover effects. Baseline d_0_-α-T concentrations were measured before each test meal (0 h). The study was also not blinded; however, personnel conducting plasma and data analyses were blinded to the intervention assignments.

Participants completed pharmacokinetic trials in a crossover manner, serving as their own control. For the primary objective, participants consumed d_x_-spinach alone (SPINACH) or concurrently with one whole egg (SPINACH + 1 EGG_0h_), two whole eggs (SPINACH + 2 EGGS_0h_), three whole eggs (SPINACH + 3 EGGS_0h_), two egg whites (SPINACH + WHITE), or vegetable oil (SPINACH + OIL). For the exploratory objective, additional test meals included d_x_-spinach with delayed egg consumption (SPINACH + EGG_3h_) and d_x_-spinach with one egg concurrently and a second at 3 h (SPINACH + EGGS_0&3h_). All study visits were conducted at the Human Nutrition Metabolic Laboratory at The Ohio State University.

Participants arrived between 06:00 and 08:00 after a 10–12 h fast and consumed the assigned test meal at time 0 h. Whole blood was collected at baseline (0 h) and at 3, 4.5, 6, 7.5, 9, 12, 24, 36, 48, and 72 h post-ingestion for determination of plasma d_x_-α-T. Participants remained in the study center for the first 12 h and consumed standardized lunch and snack immediately after the blood collections at 4.5 and 6 h, respectively, followed by dinner at 10 h (total additional α-T = 5.9 mg), to control postprandial influences on α-T pharmacokinetics. Each trial was separated by a washout of at least 1 week, and pilot testing confirmed that plasma d_x_-α-T isotopomers were undetectable by 96 h following ingestion of the standardized dose.

All study procedures were approved by The Ohio State University Institutional Review Board (Protocol No. 2019H0504; approved on 13 February 2020). The study was registered at ClinicalTrials.gov (Identifier: NCT04287816) on 22 February 2020. All participants provided written informed consent prior to enrollment. Participants initially enrolled under the original 6-arm protocol were invited to participate in the exploratory arms and provided additional informed consent if they agreed (Figure 1). Participants were provided with an honorarium for their participation.

### 2.2. Participants

Healthy adults between 18 and 65 y of age were recruited from the Columbus, Ohio, USA, area through print and electronic advertisements. Eligible participants had a BMI of 19–25 kg/m^2^ and were non-smokers with fasting chemistries within normal ranges. This included plasma total cholesterol < 240 mg/dL, triglycerides < 150 mg/dL, glucose < 100 mg/dL, hematocrit of 41–50% for men and 36–48% for women, and hemoglobin of 13.5–17.5 g/dL for men and 12.0–15.5 g/dL for women. Participants were required to have no history of gastrointestinal disorders or iron deficiency anemia, be non-users of dietary supplements for at least 1 mo, and not be using any medications known to affect lipid or glucose metabolism. Exclusion criteria included food allergies, alcohol consumption > 2 drinks per day, engagement in >7 h/wk of aerobic activity, or recent body mass change (±2 kg) during the past month. Women were excluded if they were pregnant, lactating, or had initiated or modified hormonal contraceptive use within the past 3 mo.

All screening measurements were conducted using standardized procedures. Height was measured using a wall-mounted stadiometer, and body mass was assessed using a calibrated digital scale. Waist circumference was measured at the level of the umbilicus using a non-elastic tape. Following at least 15 min of seated rest, blood pressure was measured using an automated monitor (Omron BP760; Hoffman Estates, IL, USA), and the average of the two readings was recorded. Fasting (12 h) concentrations of plasma glucose (G7517120), plasma triglyceride (T75321L), plasma total cholesterol (C7510120), and whole-blood hemoglobin (H7504120) were determined using clinical assay kits (Pointe Scientific; Canton Township, MI, USA) on a UV-2600 spectrophotometer (Shimadzu; Columbia, MD, USA). Hematocrit was measured from whole blood using ammonium heparin microhematocrit capillaries (Statspin, IRIS Sample Processing; Brea, CA, USA) after centrifugation at 14,118× *g* for 3 min using a ZIPCombo Centrifuge (LW Scientific; Lawrenceville, GA, USA) and read using a microhematocrit reader.

### 2.3. Intrinsically Labeled Spinach

Spinach was intrinsically labeled to provide a plant-based dietary source of d_x_-α-T. A total of 9.5 kg of spinach was grown hydroponically in an environmentally controlled growth chamber at The Ohio State University Department of Horticulture and Crop Sciences, with regulated light:dark cycles (16 h/8 h), with mean temperature and humidity maintained at 21 °C and 44%, respectively. Spinach was selected as the model vegetable because it is among the richest dietary sources of α-T [11], is commonly co-consumed with eggs, and has a rapid growth cycle (~6 weeks) with minimal loss of α-T following cooking and freezing [31,32].

Platypus RZ F1 seeds (Rijk Zwaan, De Lier, The Netherlands) were germinated in rockwool for approximately 1 wk and then transferred to hydroponic trays containing nutrient solution prepared with 30 atom% deuterium oxide (Sigma-Aldrich ISOTEC; St. Louis, MO, USA; 99.8% atom purity). The growth medium was enriched with essential nutrients using Hydro-Gro Vine fertilizer and calcium nitrate supplements (CropKing; Lodi, OH, USA). Plants were grown to maturity (~6 weeks), harvested, washed, pooled across batches, and mechanically chopped. To standardize preparation and improve handling, spinach was microwaved for 30 s, a process shown to minimally affect α-T content [32], vacuum-sealed, and stored at −20 °C until use. Pooling of spinach from multiple growth batches was performed to further minimize variability in α-T content and isotopic enrichment across trials. An aliquot of the pooled spinach was analyzed by LC-MS to determine α-T content and isotopomer distribution of d_x_-α-T. For each pharmacokinetic trial, participants consumed ~100 g of d_x_-spinach (118 ± 7.5 g, mean ± SEM; range: 94–134 g), which was thawed and microwaved for 60 s prior to administration, providing 5 mg d_x_-α-T in each trial. A participant was enrolled in the crossover study when sufficient d_x_-spinach (pooled from multiple harvests) was available to complete all study arms. This approach ensured that the within-subject dose was always identical between trials because the pooled labeled spinach was aliquoted into single-serving portions to deliver 5 mg α-T.

### 2.4. Test Meals and Dietary Control

Test meals were designed to evaluate the effects of egg dose, food matrix, and meal timing on d_x_-α-T. All interventions included a standardized portion of d_x_-spinach providing 5 mg of d_x_-α-T, which was consumed either alone or in combination with co-ingested foods varying in lipid content and composition. These interventions correspond to the experimental conditions described above. Whole eggs, egg whites, and vegetable oil were used to manipulate dietary fat and food matrix characteristics. Large eggs were purchased at a local grocery store (Kroger, Columbus, OH, USA), selected to ensure consistent mass (61.9 ± 0.5 g; mean ± SEM), and were prepared by hard-boiling. Whole eggs were provided intact, whereas egg whites were separated to remove yolk-derived lipids while retaining protein. Each whole egg contained approximately 0.5 mg α-T, which was confirmed by LC-MS. Soybean oil (Good & Gather; Minneapolis, MN, USA) was provided as a triglyceride-rich lipid source at a dose of 9.6 g, which matched the fat content of two whole eggs and contained approximately 0.8 mg α-T per 9.6 g.

To minimize variability in dietary intake, participants consumed a fully controlled diet for 3 d prior to each pharmacokinetic trial and during the initial 12 h of the trial period (Supplementary Table S1). The prescribed diet provided 53 ± 0.5% of total energy from carbohydrate, 16 ± 0.5% from protein, and 31 ± 0.4% from fat, and contained 7.2 ± 0.3 mg/day of α-T (~50% of the Recommended Dietary Allowance), reflecting typical dietary intake patterns [2]. All meals were prepared and provided by the study team to standardize macronutrient composition and background α-T intake across participants and study conditions.

### 2.5. LC-MS Analysis

**Figure 2:**
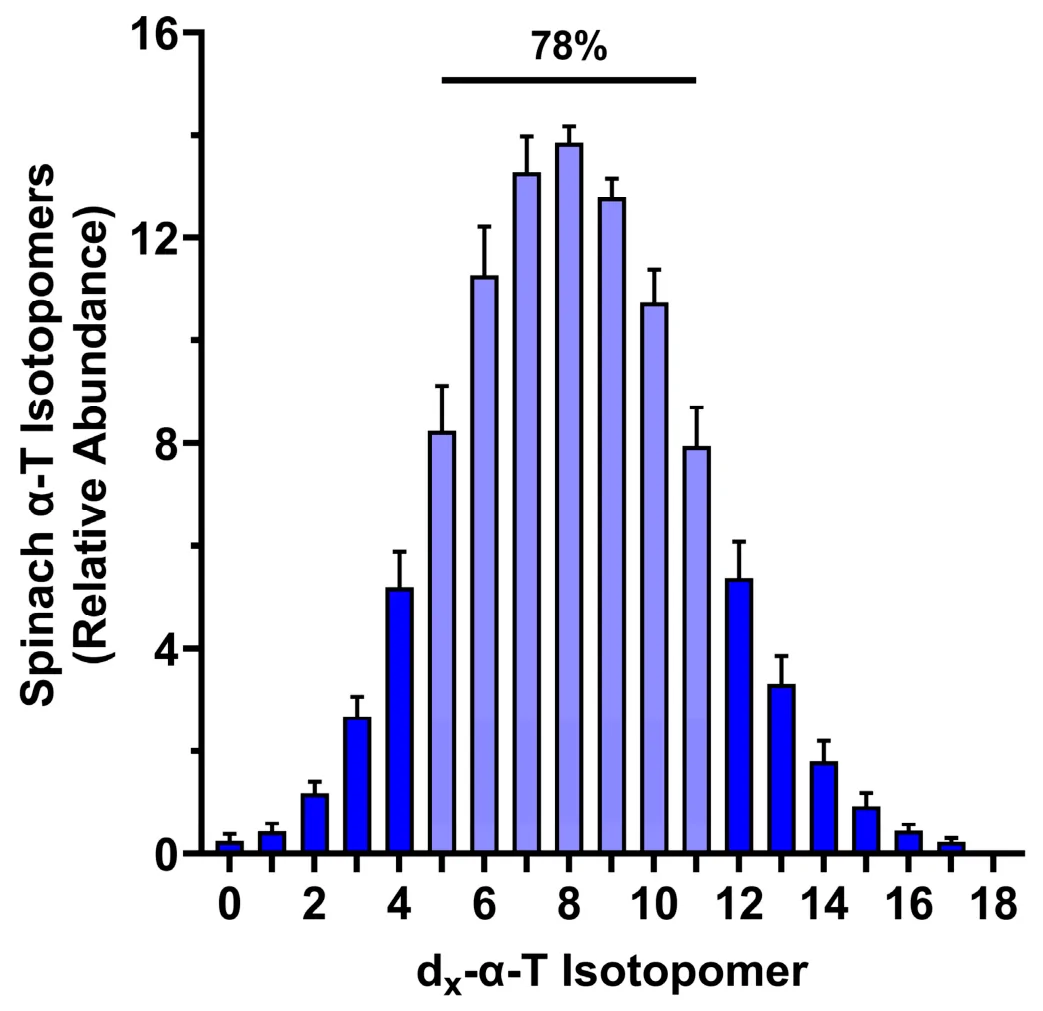
Isotopomer distribution of deuterium-labeled α-tocopherol (d_x_-α-T) in spinach grown with deuterium oxide. Unlabeled (d_0_) and deuterium-labeled (d_1_–d_17_) α-T isotopomers from spinach grown for 6 weeks under deuterium oxide conditions (30 atom%) were quantified by LC-MS using selected ion monitoring at mass-to-charge ratios of 429 for d_0_-α-T and 430–446 for d_1_- to d_17_-α-T (1 amu increments). Bars represent the relative abundance of each isotopomer, calculated as the peak area of each isotopomer relative to that of all isotopomers (d_0_–d_17_). The distribution exhibits an approximately normal distribution centered on mid-range isotopomers, with d_5_–d_11_ (light-shaded bars) accounting for 78% of total α-T. These predominant isotopomers were used to quantify plasma d_x_-α-T following the ingestion of spinach by participants. Data are means ± SEM calculated across seven independent samples of pooled, hydroponically grown spinach (i.e., biological replicates). Within each biological replicate, five spinach samples were assessed for isotopomer distribution in an independent manner (i.e., technical replicates). Values obtained from the technical replicates were averaged prior to assessing the overall variability of biological replicates.

An established LC-MS method, with minor modifications, was used to quantify labeled and unlabeled α-T in plasma and spinach [18,19,33]. Briefly, whole blood was collected into evacuated tubes containing K_2_-EDTA (Becton Dickinson; Franklin Lakes, NJ, USA) and centrifuged (2000× *g*, 15 min, 4 °C) to isolate plasma, which was aliquoted and stored at −80 °C. For analysis, 300 µL plasma or 50 mg spinach was saponified at 70 °C in alcoholic potassium hydroxide containing ascorbic acid. Samples were extracted with hexane, dried under nitrogen gas, reconstituted in methanol:ethanol (1:1), and injected onto a Shimadzu LCMS-2020 single-quadrupole mass spectrometry system equipped with an atmospheric pressure chemical ionization probe operated in negative ion mode. Isocratic separation was performed using 100% methanol at 0.3 mL/min on a Kinetex C_18_ column (100 × 2.1 mm, 2.6 µm; Phenomenex; Torrance, CA, USA) maintained at 30 °C. Selected ion monitoring was used to detect unlabeled (d_0_) and labeled (d_1_–d_17_) α-T isotopomers (*m*/*z* 429–446) and the internal standard tocol (*m*/*z* 387.3; 41 pmol injected). Unlabeled and labeled spinach and plasma α-T concentrations were calculated as peak area ratios relative to the internal standard against an external authentic α-T standard, accounting for extraction efficiency and instrument variability. Plasma d_x_-α-T concentrations were quantified from the predominant isotopomers (*m*/*z* 434–440), which were summed and adjusted to reflect the isotopomer distribution measured in spinach (Figure 2). Representative LC–MS chromatograms of plasma α-T are provided in Supplementary Figure S1. To minimize analytical variability, all samples from a given participant across study conditions were analyzed within the same analytical batch.

### 2.6. Plasma d_x_-α-T Pharmacokinetics

Plasma d_x_-α-T pharmacokinetic parameters were estimated using non-compartmental analysis as we described [18,19,33]. For each participant and trial, the maximum plasma concentration (C_max_) and time to maximum concentration (T_max_) were determined from the concentration-time profiles. The area under the plasma concentration–time curve was calculated using the linear trapezoidal method over 0–12 h (AUC_0–12h_) to assess the absorptive phase and over 0–72 h (AUC_0–72h_) to assess overall bioavailability. The elimination rate constant (K_e_) was estimated from the slope of the log-linear portion of the concentration–time curve during the terminal elimination phase. The elimination half-life (t_1/2_) was calculated as ln(2)/K_e_. Fractional absorption of d_x_-α-T (% of ingested dose) was estimated from the plasma d_x_-α-T concentration extrapolated back to time zero from the linear regression analysis. Total blood volume was estimated using the Nadler equation based on sex, height, and body weight [34]. Plasma volume was calculated as blood volume × (1 − hematocrit). The extrapolated concentration at time zero and estimated plasma volume were used to calculate the amount of absorbed d_x_-α-T in circulation, which was expressed relative to the administered dose. This approach provides an estimate of relative absorption under controlled conditions consistent with prior pharmacokinetic α-T studies [19,35].

### 2.7. Statistical Analysis

Plasma d_x_-α-T AUC_0–72h_ was the primary outcome and served as the basis for power calculations. Sample size estimates were derived from a previously published study demonstrating a dose-dependent increase in α-T bioavailability with dietary fat [18]. Using the smallest observed difference in AUC_0–72h_ between low-fat conditions, a sample size of five participants was determined to provide >90% power (α = 0.05).

All analyses were conducted per protocol. d_0_-α-T concentrations at baseline (0 h for each trial) were compared across test meal conditions to assess potential differences in α-T status upon trial initiation. All data (means ± SEM) were analyzed using GraphPad Prism version 11.0.0 for Windows, GraphPad Software, Boston, MA, USA). Statistical significance was set to *p* ≤ 0.05. Prior to analysis, data were evaluated for homogeneity of variance using the Brown–Forsythe test. When necessary, data were log-transformed to meet statistical assumptions of parametric testing. Pre-specified between-treatment comparisons were conducted to evaluate: (i) the dose-dependent effects of eggs on d_x_-α-T pharmacokinetics (SPINACH, SPINACH + 1 EGG_0h_, SPINACH + 2 EGGS_0h_, SPINACH + 3 EGGS_0h_); (ii) food matrix effects (SPINACH + 2 EGGS_0h_, SPINACH + WHITE, SPINACH + OIL); and (iii) meal timing effects (SPINACH, SPINACH + 1 EGG_0h_, SPINACH + EGG_3h_, SPINACH + EGGS_0&3h_).

For the primary objective, dose-dependent and food matrix comparisons were analyzed using linear mixed-effects models accounting for within-subject repeated measures. Pairwise comparisons were performed using the Holm–Sidak post hoc test to control family-wise error while maintaining statistical power for pre-specified comparisons. For the exploratory objective (meal timing), analyses were performed separately using linear mixed-effects models followed by Fisher’s least significant difference test when a significant main effect was observed, without adjustment for multiple comparisons given the exploratory nature of these analyses.

## 3. Results

### 3.1. Participants and Diet

Eight healthy adults were enrolled and completed the primary study without any adverse effects. All participants had normal BMI, blood pressure, and fasting metabolic parameters (Table 1). Participants consumed a standardized diet for three days prior to each trial and during the first 12 h post-intervention, providing 2495 ± 28 kcal/day, comprising 333.2 ± 5.8 g carbohydrate, 99.6 ± 1.6 g protein, and 87.6 ± 1.7 g fat, and supplying 7.4 ± 0.3 mg/day of α-T. There were no significant differences between trials for energy, macronutrient, or α-T intakes (*p* > 0.05).

### 3.2. Dose-Dependent Effects of Egg Consumption on Spinach-Derived d_x_-α-T

**Figure 3:**
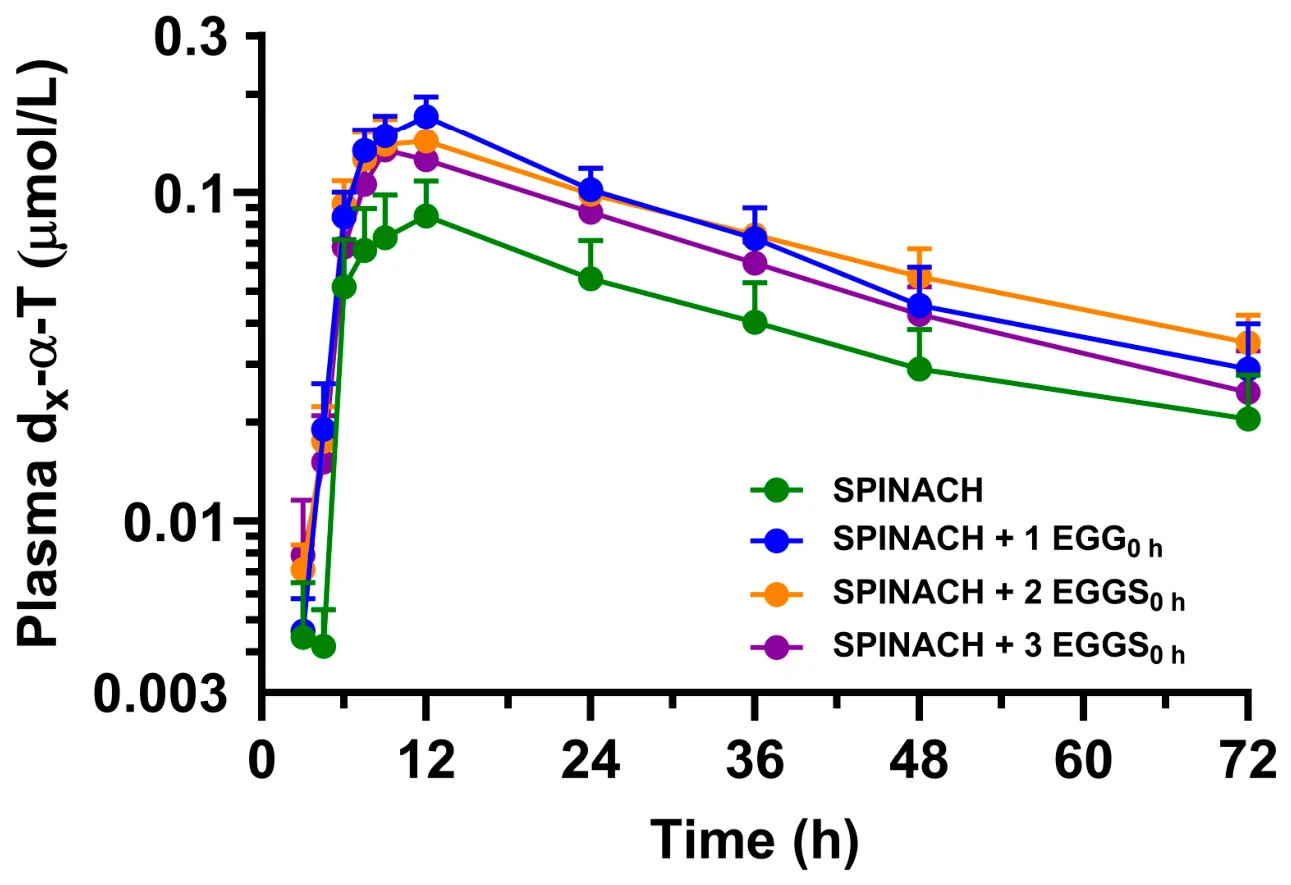
Plasma concentration-time curves of deuterium-labeled α-tocopherol (d_x_-α-T) in healthy participants following ingestion of spinach with 0–3 eggs. Participants consumed a standardized dose of d_x_-α-T (5 mg) from intrinsically labeled spinach alone (SPINACH) or concurrently with 1, 2, or 3 whole eggs. Plasma d_x_-α-T concentrations were measured by LC–MS in samples collected at baseline (0 h) and at specified time points over 72 h post-ingestion. Co-ingestion with eggs increased plasma d_x_-α-T concentrations relative to spinach alone, with no apparent dose-dependent effect. The *y*-axis is displayed on a logarithmic scale. Data are means ± SEM (*n* = 8).

We first evaluated whether egg dose influences the bioavailability of d_x_-α-T from intrinsically labeled d_x_-spinach. Plasma concentration-time curves are shown in Figure 3, with pharmacokinetic parameters summarized in Table 2. Baseline plasma α-T concentrations were within the adequate range (>12 µmol/L) [2] and did not differ between trials (*p* > 0.05). Compared with SPINACH alone, concurrent co-ingestion of eggs significantly increased d_x_-α-T early appearance and overall bioavailability, as reflected by higher AUC_0–12h_ and AUC_0–72h_ values (*p* ≤ 0.05). However, there were no differences among the egg-containing treatments, indicating that increasing egg dose beyond one egg did not further enhance d_x_-α-T bioavailability. A similar pattern was observed for C_max_, which increased with egg co-ingestion (*p* ≤ 0.05 vs. SPINACH) but did not differ across egg doses (*p* > 0.05). In contrast, T_max_ did not differ between treatments (*p* > 0.05). Fractional absorption was low following SPINACH alone (2.39 ± 0.60%) and increased with egg co-ingestion (*p* ≤ 0.05) but did not differ between egg doses. Likewise, no differences were observed in elimination rate constant (K_e_) or half-life (t_1/2_) across treatments (*p* > 0.05). Collectively, these findings indicate that egg co-ingestion, rather than egg dose, is the primary determinant of increased d_x_-α-T bioavailability.

### 3.3. Food Matrix Effects on α-T Bioavailability

**Figure 4:**
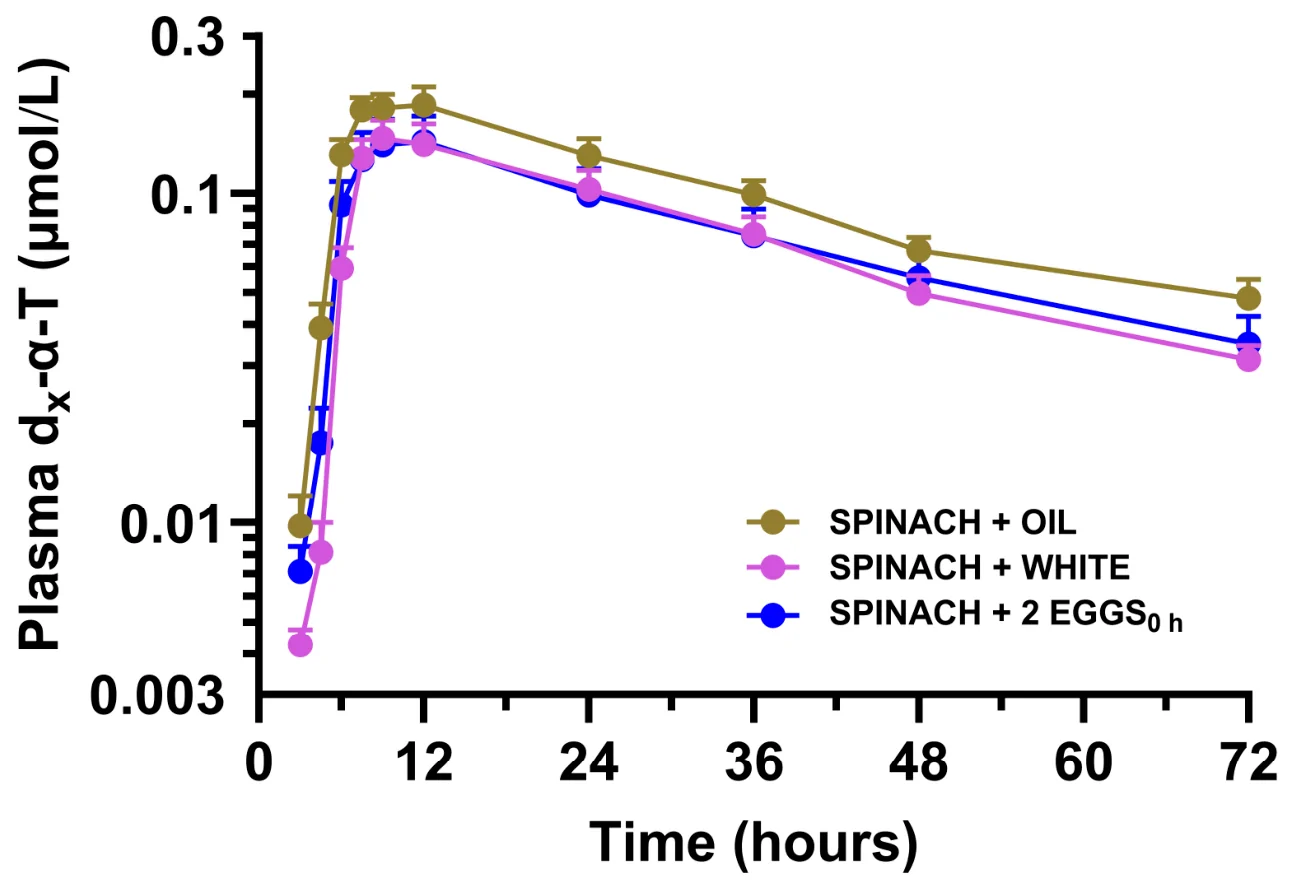
Plasma concentration–time curves of deuterium-labeled α-tocopherol (d_x_-α-T) in healthy participants following ingestion of spinach across food matrix conditions. Participants consumed a standardized dose of d_x_-α-T (5 mg) from intrinsically labeled spinach concurrently with 2 whole eggs (SPINACH + 2 EGGS_0h_), 2 egg whites (SPINACH + WHITE), or 9.6 g vegetable oil (SPINACH + OIL). Plasma d_x_-α-T concentrations were measured by LC–MS in samples collected at baseline (0 h) and at time intervals over 72 h post-ingestion. Plasma d_x_-α-T responses were comparable across food matrix conditions. The *y*-axis is displayed on a logarithmic scale. Data are means ± SEM (*n* = 8).

We next assessed whether food matrix influences the bioavailability of d_x_-α-T. Plasma concentration-time responses are shown in Figure 4, with corresponding pharmacokinetic parameters summarized in Table 2. Among test meal conditions matched for total fat content, no differences were observed between SPINACH + 2 EGGS_0h_ and SPINACH + OIL for AUC_0–12h_, AUC_0–72h_, C_max_, T_max_, fractional absorption, elimination rate (K_e_), or half-life (t_1/2_) (*p* > 0.05 for all variables). Similarly, no differences were observed between the fat-containing test meals (SPINACH + 2 EGGS_0h_ and SPINACH + OIL) and the egg protein-based condition (SPINACH + WHITE) for any pharmacokinetic parameter (*p* > 0.05). Collectively, these findings indicate that neither the phospholipid-rich matrix of eggs nor dietary fat per se enhanced bioavailability of d_x_-α-T relative to a protein-based matrix under these conditions.

### 3.4. Meal Timing Effects on α-T Bioavailability

**Figure 5:**
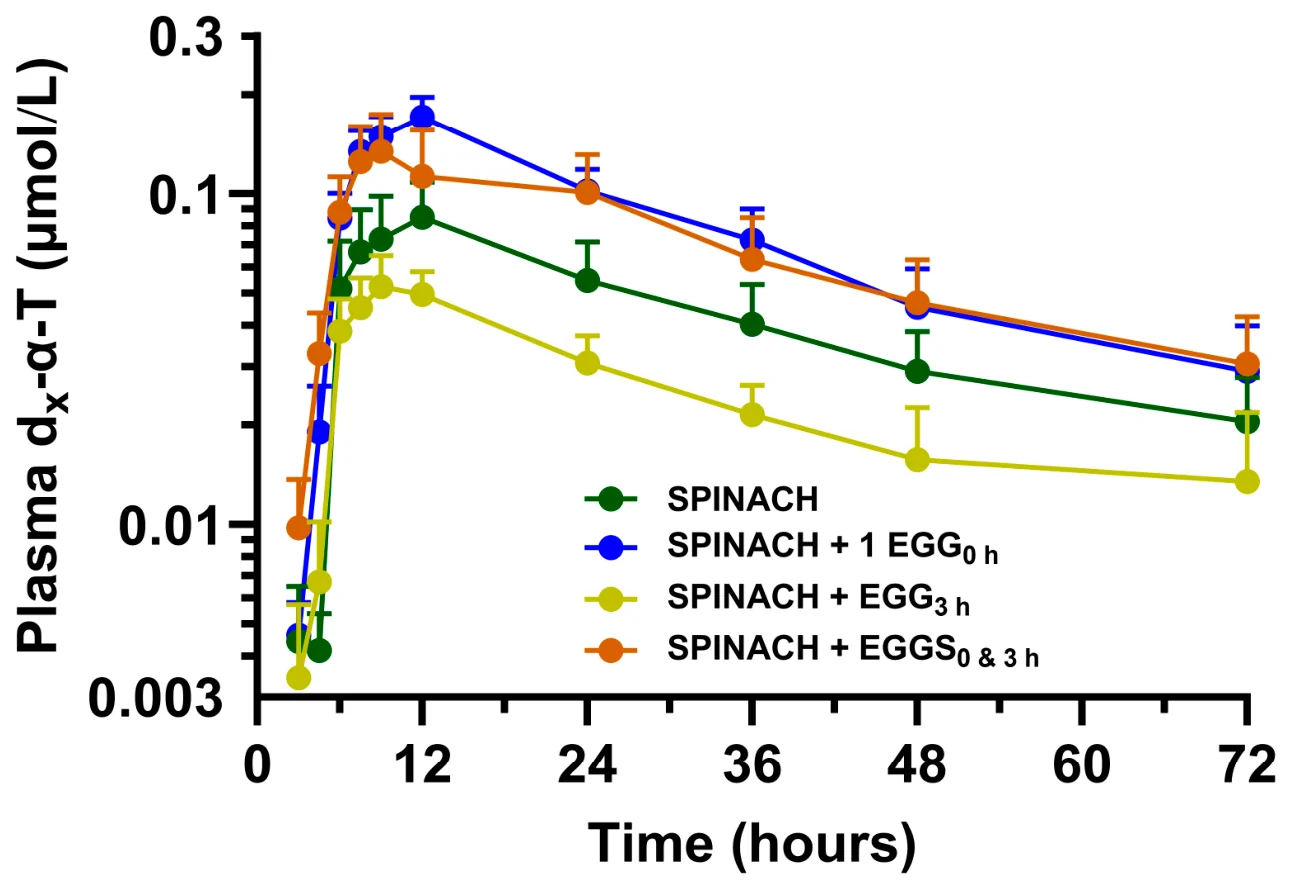
Plasma concentration–time curves of deuterium-labeled α-tocopherol (d_x_-α-T) in healthy participants following ingestion of spinach across meal-timing conditions. Participants consumed a standardized dose of d_x_-α-T (5 mg) from intrinsically labeled spinach alone (SPINACH) or with eggs consumed either concurrently (SPINACH + 1 EGG_0h_), 3 h after spinach ingestion (SPINACH + 1 EGG_3h_), or in split doses at 0 h and 3 h (SPINACH + EGGS_0&3h_). Plasma d_x_-α-T concentrations were measured by LC–MS in samples collected at baseline (0 h) and at timed intervals over 72 h post-ingestion. Delayed egg consumption attenuated plasma d_x_-α-T responses relative to concurrent ingestion. The *y*-axis is displayed on a logarithmic scale. Data are means ± SEM (*n* = 5).

Finally, we examined whether the timing of egg consumption relative to spinach intake influences d_x_-α-T bioavailability. A subset of participants (*n* = 2M/3F; 27.4 ± 1.8 y; 20.9 ± 0.7 kg/m^2^) completed these exploratory trials (Figure 5; Table 2). No statistically significant differences in baseline characteristics were observed between this substudy population and the overall study population (*p* = 0.29–0.87). Data from the SPINACH and SPINACH + 1 EGG_0h_ trials from these same participants were reused for comparison with the additional meal timing test conditions, allowing all four treatments to be evaluated within-subject. Consistent with the primary analyses, concurrent co-ingestion of eggs with spinach increased both d_x_-α-T AUC_0–12h_ and AUC_0–72h_ relative to SPINACH alone (*p* ≤ 0.05). In contrast, delaying egg consumption did not improve bioavailability, as AUC_0–12h_ and AUC_0–72h_ for SPINACH + 1 EGG (3 h) did not differ from SPINACH and were significantly lower than those observed with concurrent egg intake (*p* ≤ 0.05). Providing eggs as a split dose at 0 h and then again at 3 h did not further enhance d_x_-α-T bioavailability relative to concurrent intake in SPINACH + 1 EGG_0h_ (*p* > 0.05). Similar patterns were observed for C_max_ and fractional absorption, whereas no differences were observed for T_max_, elimination rate (K_e_), or half-life (t_1/2_) across the four test meals (*p* > 0.05). Collectively, these findings demonstrate that enhancement of d_x_-α-T bioavailability by eggs requires concurrent co-ingestion with spinach and is attenuated when egg consumption is delayed.

## 4. Discussion

This study demonstrates that the bioavailability of plant-derived α-T is determined primarily by the timing of co-ingested foods rather than the amount or type of dietary fat. Using intrinsically labeled spinach to deliver α-T in an intact food matrix, we show that concurrent ingestion with eggs significantly enhanced α-T bioavailability, whereas delaying egg consumption prevented this effect. We attribute this enhancement to increased absorption rather than delayed elimination, as post-absorptive kinetics were largely unchanged across treatments. Increasing egg dose did not further increase bioavailability, indicating that the presence of a co-ingested food, rather than the quantity of lipid, is sufficient to achieve maximal absorption under these conditions. α-T bioavailability was also similar when spinach was consumed with phospholipid-rich eggs, triglyceride-rich oil, or fat-free egg whites, demonstrating that neither dietary fat content nor lipid class is the primary determinant of α-T absorption. These findings show that α-T bioavailability from spinach is poor when consumed alone but can be significantly improved by co-ingesting other foods at the same time. This limitation is particularly important given the role of α-T in protecting cell membranes from oxidative damage and maintaining antioxidant defense systems. Together, these results challenge the prevailing view that dietary fat is required to enhance vitamin E bioavailability and instead identify co-ingestion, particularly its timing, as the critical factor. Given the emphasis on plant-forward dietary patterns, these findings have important implications for improving vitamin E status and support strategies to enhance antioxidant protection through optimized dietary practices.

Existing evidence indicates that dietary fat can enhance α-T absorption, although findings across studies have been inconsistent [18,19,36]. Several studies using supplemental or extrinsically labeled α-T report increased bioavailability with co-ingested fat [18,36], whereas others show little or no effect of fat content on α-T absorption [19,20]. These discrepancies have been attributed to differences in the chemical form of α-T, the administered dose, and the food matrix in which α-T is delivered. In particular, most studies have relied on esterified forms of α-T or supplement-based delivery systems [18,36], which do not reflect the structural and physiological constraints of plant-derived α-T embedded within cellular membranes. In addition, few studies have systematically examined the combined effects of food matrix and meal timing on α-T bioavailability [20,36,37]. As a result, it remains unclear whether dietary fat itself is the primary determinant of α-T absorption from plant foods or whether other factors related to food co-ingestion and digestive timing play a more important role. The present study addresses this gap by using intrinsically labeled spinach to quantify plant-derived α-T bioavailability while independently evaluating the effects of dose, matrix, and timing of co-ingested foods.

The observed low bioavailability of α-T from spinach in the present study is consistent with known limitations of plant-based food matrices [14,38], in which α-T is embedded within chloroplast membranes and not readily released during digestion [38]. This limitation can be overcome by certain food preparation methods [39]. In support, when d_x_-collard greens were steamed for 8–12 min and pureed prior to ingestion, d_x_-α-T absorption from the collard greens co-ingested with a dairy food-based breakfast (450 kcal) with modest fat and protein was estimated at 24% ± 16% [35]. However, our approach of minimally microwaving spinach, primarily for preservation and food safety, reflected a translational focus, as spinach is often consumed with limited cooking. Another factor potentially contributing to low α-T is the low intrinsic lipid content of leafy vegetables, which limits micelle formation, thereby restricting solubilization and uptake of α-T in the small intestine. Under these conditions, only a fraction of the ingested α-T becomes bioaccessible, which likely explains the low circulating concentrations observed when spinach was consumed alone. Co-ingestion with other foods likely overcomes these plant matrix effects, at least in part, by promoting digestive processes that enhance α-T absorption. Both dietary fat and protein have been shown to stimulate the release of cholecystokinin, which increases bile secretion and supports the formation of mixed micelles that mediate uptake at the enterocyte brush border of fat-soluble compounds [26,40,41]. In our study, the similar enhancement of α-T bioavailability observed with eggs, vegetable oil, and egg whites suggests that this effect is driven by stimulation of digestive processes rather than by the provision of lipid as phospholipid-rich eggs or triglyceride-rich vegetable oil. These findings indicate that co-ingestion enhances the bioaccessibility of α-T from a plant matrix to an absorbable form independent of dietary lipid content.

The absence of a dose-dependent response to egg intake in our study suggests that α-T absorption plateaus once conditions supporting micelle formation are achieved. In our study, increasing egg intake served to increase lipid from a phospholipid-rich food source. However, after consumption of a single egg, stimulation of biliary and pancreatic secretions was likely sufficient to solubilize and facilitate the absorption of bioaccessible α-T, thereby precluding any additional benefit from higher egg intake. This is consistent with studies using non-esterified α-T, which report no further increase in bioavailability with increasing dietary fat (0.2–7.9 g) when α-T (15 mg) is co-consumed at a fixed dose as a supplement outside of the food matrix [19]. In addition, very high fractional absorption occurred when non-esterified α-T is consumed at low doses (<1 mg) in supplement form, further indicating that α-T absorption is not inherently limited by the absence of dietary fat under these conditions [42]. In contrast, studies demonstrating fat-dependent increases in α-T absorption have largely used α-tocopheryl acetate at higher doses, where hydrolysis and increased requirements for micellarization may render absorption more sensitive to the amount of co-ingested lipid [36]. In support, simulated digestion studies indicate that fat-dependence of non-esterified α-T occurs at higher doses, when the micellarization capacity of the digestive system likely becomes limiting [19]. Together, these findings indicate that the influence of dietary lipid on α-T bioavailability is dependent on both the chemical form and likely the propensity for luminal bioaccessibility of α-T. Here, the dose of α-T from spinach was unlikely to exceed micellarization capacity, such that maximal absorption could be achieved with minimal co-ingestion. Under these conditions, increasing egg intake did not potentiate bioavailability, likely because micellarization and absorption were no longer rate-limiting.

Our observed lack of a food matrix effect further supports the conclusion that α-T absorption is not dependent on the lipid amount or composition of co-ingested foods. Although eggs provide a phospholipid-rich matrix and vegetable oil provides triglyceride-rich lipid, both resulted in similar α-T bioavailability, indicating that lipid class does not influence spinach-derived α-T absorption. Notably, α-T bioavailability was also enhanced to levels no different from those observed in vegetable oil- or egg-containing trials when spinach was consumed with fat-free egg whites, demonstrating that the presence of lipid is not required to stimulate α-T absorption. Our findings suggest that α-T bioavailability from these different food matrices is attributable to the stimulation of digestive processes rather than the direct contribution of dietary lipid. Indeed, both fatty acids and amino acids stimulate cholecystokinin [26,43], which promotes bile secretion and micelle formation. Our findings suggest that egg whites may have enhanced α-T absorption through protein-mediated stimulation of these pathways, despite the absence of lipid. Thus, co-ingestion of foods with or without dietary lipid enhanced α-T bioavailability, likely by facilitating digestive responses that increase α-T bioaccessibility and subsequent intestinal absorption.

The meal timing-dependent effects observed in our study support that a limited window exists for α-T absorption following food ingestion. Concurrent consumption of spinach with eggs enhanced α-T bioavailability, whereas delaying egg intake by 3 h limited bioavailability to the extent observed from spinach alone despite the later availability of eggs to support digestive processes. Thus, most bioaccessible α-T is likely taken up rapidly at enterocytes during the postprandial period, and that delayed co-ingestion occurs outside this effective absorption window. This interpretation is consistent with prior work demonstrating that vitamin E absorption depends on the simultaneous availability of α-T and digestive processes. In a crossover study using dual isotopes, delayed lipid intake altered α-T kinetics and delayed peak plasma concentrations, supporting the importance of temporal alignment for absorption [20]. Together, these data suggest that effective α-T absorption requires co-ingestion with foods that stimulate bile secretion at the time spinach-derived α-T becomes bioaccessible in the intestinal lumen. Although these timing effects were evaluated in a subset of participants and should be interpreted in an exploratory manner, their consistency with established digestive physiology supports their biological relevance. Under these conditions, delayed co-ingestion does not maintain α-T bioavailability, as absorption occurs predominantly during the more immediate postprandial period.

Our study provides a direct assessment of α-T bioavailability from a plant food using intrinsic stable isotope labeling, which offers important advantages over prior approaches [44]. Most studies of α-T absorption have relied on extrinsically labeled supplements that are often esterified, thus lacking the chemical structure present naturally in plant foods where α-T is biosynthesized. Other studies have also considered α-T bioavailability from foods or supplements lacking isotopic labeling [30,45], which do not allow for distinguishing between endogenous and dietary sources of α-T. By intrinsically labeling spinach, we were able to directly assess plant-derived α-T bioavailability in circulation under controlled dietary conditions. Few studies have applied intrinsic labeling to examine α-T bioavailability from plant foods. In a crossover study using intrinsically labeled collard greens, α-T kinetic parameters were generally similar to those observed in the present study when spinach was co-ingested with different test foods [35]. These similarities suggest that the fundamental processes regulating α-T absorption and distribution are consistent across leafy green vegetables. However, differences in estimated fractional absorption between studies (24% [35] vs. 4–5% here) likely reflect variation in food preparation, plant matrix disruption, and delivery conditions, which would be expected to influence plant tissue α-T release and downstream absorptive processes [39]. These findings highlight the importance of studying α-T bioavailability within intact food matrices, ideally with intrinsic labeling approaches, to better understand how dietary and physiological factors influence the absorption of α-T from plant foods.

An important consideration in interpreting our findings is that the administered α-T dose does not reflect the fraction that is bioaccessible for absorption. In spinach, α-T is localized within chloroplast membranes [46] and must be released during digestion before it can be incorporated into micelles and subsequently absorbed by enterocytes [13]. Consequently, only a portion of the ingested α-T is available for brush-border enterocyte uptake, which likely explains the relatively low fractional absorption observed across treatments. In addition, the estimated amount of d_x_-α-T in plasma relative to the administered dose reflects fractional absorption rather than total systemic absorption. Studies using radiolabeled tracers and multi-compartment modeling or “gold standard” dual-isotope methods have reported higher estimates of α-T absorption [20], suggesting that the true absorption of α-T may be higher than the values reported here. Despite this limitation, the consistent application of this method across all treatments allows for valid comparisons of relative bioavailability. Together, the apparent low absorption of α-T in this study is likely driven by limited bioaccessibility of α-T from the spinach matrix. Under these conditions, co-ingestion increases the proportion of bioaccessible α-T that is absorbed but does not fully overcome the limited release of α-T from the spinach matrix.

Our findings have important implications for improving the status of α-T, a vitamin E isoform possessing established antioxidant functions, in the context of plant-forward dietary patterns. Although leafy green vegetables such as spinach are recognized as nutrient-dense sources of α-T [11], their contribution to vitamin E status is limited by both low intake and poor bioavailability when consumed alone [12,13,14]. Our evidence supports that this limitation can be addressed, at least in part, through food-pairing strategies, whereby co-ingestion with fat- or protein-rich foods enhances plant-derived α-T absorption. Our findings also indicate that dietary recommendations focused on increasing vegetable intake may not fully address vitamin E inadequacy [5] without consideration of how these foods are consumed. Incorporating guidance on co-ingestion, particularly the timing of consumption with other foods, represents a practical approach to improve α-T bioavailability from plant foods. Such strategies are warranted given the widespread prevalence of suboptimal vitamin E intake [4,47,48].

This study has several strengths that enhance the interpretation of these findings. Our use of intrinsic stable isotope labeling allowed for direct quantification of plant-derived α-T bioavailability, eliminating confounding from α-T present in co-ingested foods and providing a physiologically relevant assessment within an intact food matrix. The crossover design, with each participant serving as their own control, minimized interindividual variability while improving sensitivity for detecting treatment effects. Likewise, our use of a controlled basal diet likely reduced variability in nutrient intake that potentially influences α-T pharmacokinetics. We also acknowledge several limitations, including a relatively small sample, which should be considered when generalizing these findings. However, the intrinsic stable isotope approach substantially improves sensitivity for detecting treatment effects, and our sample size calculation indicated that only five participants would be sufficient to detect the primary outcome, supporting the adequacy of the enrolled sample. In addition, the lack of randomization of the meal timing trials reflects logistical constraints and necessitates cautious interpretation of these results. However, our controlled diet, standardized on-site meal administration, and comparable baseline α-tocopherol concentrations across study conditions likely minimized the potential impact of the non-randomized design on the interpretation of these findings. In addition, we suggest that the enhanced α-T bioavailability observed with egg white co-ingestion may have resulted from protein-mediated stimulation of cholecystokinin release, bile secretion, and micelle formation. However, these processes were not measured in the present study, and the proposed mechanism requires confirmation in future studies. Finally, because biomarkers of oxidative stress or antioxidant activity were not measured, the present findings should not be interpreted as direct evidence of enhanced antioxidant protection. Rather, the study demonstrates improved bioavailability of α-T, which is a prerequisite for its biological antioxidant functions. Despite these limitations, the consistency of findings across treatments and their alignment with established digestive physiology support the validity of the conclusions.

## 5. Conclusions

In conclusion, these findings demonstrate that α-T bioavailability from spinach is limited when consumed alone but can be significantly improved through co-ingestion with other foods. The effectiveness of this strategy depends primarily on the timing of co-ingestion, with concurrent intake required to support absorption during the immediate postprandial period. In contrast, increasing the amount or altering the lipid composition of co-ingested foods did not further enhance bioavailability, indicating that digestive stimulation rather than dietary lipid quantity is the primary factor governing absorption under these conditions. Overall, these findings advance a practical framework for improving α-T bioavailability from plant foods and indicate that dietary recommendations should extend beyond selection of α-T-rich foods to include how and when foods are consumed. Food-pairing strategies that align nutrient availability with host digestion may help improve vitamin E-dependent antioxidant activity and enhance the nutritional benefits of plant-based diets. However, whether improved bioavailability translates into greater antioxidant activity requires further investigation. These findings should also be interpreted within the context of a controlled pharmacokinetic study and require confirmation in larger populations.

## Figures and Tables

**Table 1:** Baseline participant characteristics ^1^.

Parameter	Mean ± SEM
Age (y)	27.9 ± 1.6
BMI (kg/m^2^)	21.8 ± 0.7
Waist Circumference (cm)	74.9 ± 1.5
Systolic Blood Pressure (mmHg)	109.6 ± 2.9
Diastolic Blood Pressure (mmHg)	70.8 ± 1.9
Blood Hemoglobin (g/dL)	13.3 ± 0.3
Blood Hematocrit (%)	43.2 ± 1.0
Total Cholesterol (mmol/L)	4.19 ± 0.17
Triglyceride (mmol/L)	0.57 ± 0.05
Total Lipid (mmol/L)	4.75 ± 0.20
Glucose (mmol/L)	4.81 ± 0.14
d_0_-α-Tocopherol (µmol/L) ^2^	20.1 ± 1.3

^1^ Values are mean ± SEM (*n* = 8; 3 men and 5 women). Total lipids represent the sum of total cholesterol and triglyceride. ^2^ Baseline d_0_-α-tocopherol concentrations are reported as participants’ mean α-tocopherol concentration at baseline (0 h) for all test meal conditions.

**Table 2:** Pharmacokinetic parameters of plasma d_x_-α-T following ingestion of d_x_-spinach under different test conditions ^1,2,3^.

Treatment	Baseline d_0_-α-T (µmol/L)	d_x_-α-T AUC_0–12h_ (µmol/L × h)	d_x_-α-T AUC_0–72h_ (µmol/L × h)	d_x_-α-T C_max_ (µmol/L)	d_x_-α-T T_max_ (h)	d_x_-α-T Absorption (% Dose)	d_x_-α-T K_e_ (1/h)	R^2^	d_x_-α-T t_1/2_ (h)
**Primary Study: Dose Effects**									
SPINACH	19.9 ± 1.4	0.49 ± 0.16 ^a^	2.90 ± 0.88 ^a^	0.09 ± 0.02 ^a^	9.64 ± 0.86	2.39 ± 0.60 ^a^	0.035 ± 0.008	0.96 ± 0.01	25.4 ± 4.5
SPINACH + 1 EGG_0h_	19.1 ± 1.3	0.96 ± 0.13 ^b^	5.24 ± 1.00 ^b^	0.18 ± 0.03 ^b^	10.10 ± 0.55	4.93 ± 0.62 ^b^	0.035 ± 0.005	0.95 ± 0.02	22.5 ± 3.3
SPINACH + 2 EGGS_0h_	20.1 ± 1.4	0.90 ± 0.17 ^b^	5.18 ± 1.06 ^b^	0.15 ± 0.03 ^b^	10.90 ± 0.55	3.99 ± 0.71 ^b^	0.028 ± 0.003	0.97 ± 0.01	26.9 ± 2.3
SPINACH + 3 EGGS_0h_	20.0 ± 1.3	0.79 ± 0.13 ^b^	4.39 ± 0.69 ^b^	0.14 ± 0.02 ^b^	9.94 ± 0.63	4.41 ± 0.72 ^b^	0.035 ± 0.007	0.97 ± 0.01	24.3 ± 3.7
**Primary Study: Matrix Effects**									
SPINACH + 2 EGGS_0h_	20.1 ± 1.4	0.90 ± 0.17	5.18 ± 1.06	0.15 ± 0.03	10.90 ± 0.55	3.99 ± 0.71	0.028 ± 0.003	0.97 ± 0.01	26.9 ± 2.3
SPINACH + WHITE	21.4 ± 2.0	0.84 ± 0.10	5.10 ± 0.65	0.15 ± 0.02	9.94 ± 0.63	4.07 ± 0.65	0.028 ± 0.001	0.98 ± 0.01	26.8 ± 1.6
SPINACH + OIL	19.7 ± 1.2	1.23 ± 0.12	6.88 ± 0.74	0.20 ± 0.02	9.38 ± 0.79	4.91 ± 0.59	0.024 ± 0.002	0.96 ± 0.02	30.5 ± 2.1
**Exploratory Study: Timing Effects**									
SPINACH	18.9 ± 2.1	0.32 ± 0.16 ^B^	2.13 ± 1.02 ^BC^	0.06 ± 0.03 ^BC^	9.30 ± 1.10	1.96 ± 0.76 ^B^	0.037 ± 0.012	0.96 ± 0.02	25.0 ± 7.2
SPINACH + 1 EGG_0h_	19.3 ± 2.1	0.92 ± 0.20 ^A^	5.36 ± 1.52 ^A^	0.18 ± 0.04 ^A^	10.80 ± 0.74	4.47 ± 0.84 ^A^	0.031 ± 0.004	0.93 ± 0.03	24.7 ± 4.3
SPINACH + 1 EGG_3h_	20.9 ± 2.3	0.33 ± 0.08 ^B^	1.67 ± 0.45 ^C^	0.06 ± 0.01 ^C^	10.20 ± 0.74	1.63 ± 0.26 ^B^	0.046 ± 0.009	0.97 ± 0.01	16.9 ± 2.5
SPINACH + EGGS_0&3h_	19.8 ± 2.1	0.84 ± 0.23 ^A^	4.88 ± 1.53 ^AB^	0.14 ± 0.04 ^AB^	9.90 ± 0.90	4.72 ± 0.57 ^A^	0.028 ± 0.005	0.98 ± 0.01	26.1 ± 3.1

^1^ Bolding in the table denotes pre-defined primary and exploratory objectives of the crossover clinical trial examining α-T pharmacokinetics and bioavailability in response to test meals of varying composition. ^2^ Abbreviations: AUC, area under the plasma-time concentration curve; C_max_, maximum concentration; d_0_, unlabeled; d_x_, deuterium-labeled; k_e_, elimination rate constant; R^2^, goodness-of-fit of the semi-log time-concentration curve for d_x_-α-T during elimination phase; t_1/2_, half-life; T_max_, time to maximum concentration. ^3^ Data are means ± SEM. Statistical analyses were performed using linear mixed-effects models accounting for within-subject repeated measures followed by Holm–Šidák (primary study; *n* = 8) and Fisher’s least significant difference (exploratory study; *n* = 5) post hoc tests. For the dose effect study, values within a column not sharing the same lowercase superscript letter are significantly different (*p* ≤ 0.05). For the matrix effect study, no statistically significant effects were observed (*p* > 0.05). In the timing effects study, values within a column not sharing the same uppercase superscript letter are significantly different (*p* ≤ 0.05).

## Data Availability

Due to ethical and confidentiality constraints, the human participant data cannot be publicly shared. Limited, anonymized datasets may be requested from the corresponding author, subject to ethical approval.

## Supplementary Materials

The following supporting information can be downloaded at: https://www.mdpi.com/article/10.3390/antiox15091076/s1, Figure S1: Representative LC-MS chromatograms of α-tocopherol (α-T) in plasma collected 12 h after ingestion of intrinsically deuterium-labeled spinach (d_x_-spinach) with two eggs; Table S1: Food and nutrient composition of controlled diets administered to participants during the 3 days prior to and the initial 12 h of each pharmacokinetic trial.

## Abbreviations

The following abbreviations are used in this manuscript:

α-T	α-Tocopherol
AUC	Area under the plasma concentration–time curve
BMI	Body mass index
C_max_	Maximum plasma concentration
d_0_	unlabeled
d_x_	Deuterium-labeled
K_e_	Elimination rate constant
LC-MS	Liquid chromatography–mass spectrometry
SEM	Standard error of the mean
t_1/2_	Half-life
T_max_	Time to maximum plasma concentration
