# Supplementary material for: Co-Ingestion Timing, Rather than Food Matrix or Dose, Determines α-Tocopherol Bioavailability from Intrinsically Labeled Spinach in Healthy Adults: A Crossover Pharmacokinetic Study
Source: Antioxidants (Basel). 2026 Aug 28;15(9):1076. doi: 10.3390/antiox15091076 (PMC13603847; doi:10.3390/antiox15091076)
Supplement: Supplementary file 1 [file antioxidants-15-01076-s001.zip › antioxidants-4424760-supplementary.pdf]

Supplementary Materials

Co-Ingestion Timing, Rather than Food Matrix or Dose, Determines α-Tocopherol Bioavailability from Intrinsically Labeled Spinach in Healthy Adults: A Crossover Pharmacokinetic Study

Shahabeddin Rezaei [1,†], Sisi Cao [1,†], Min Zeng [1], Maria L. Antonius [1], Jillian T. Pierson [1], Ariana H. Bond [1], Allyson Q. McHenry [1], Joshua J. Blakeslee [2] and Richard S. Bruno [1,*]

[1] Human Nutrition Program, The Ohio State University, Columbus, OH 43210, USA; rezaei.17@osu.edu (S.R.); pierson.150@buckeyemail.osu.edu (J.T.P.)
[2] Department of Horticulture and Crop Science, The Ohio State University, Columbus, OH 43210, USA
* Correspondence: bruno.27@osu.edu; Tel.: +1-614-292-5522
† These authors contributed equally to this work.

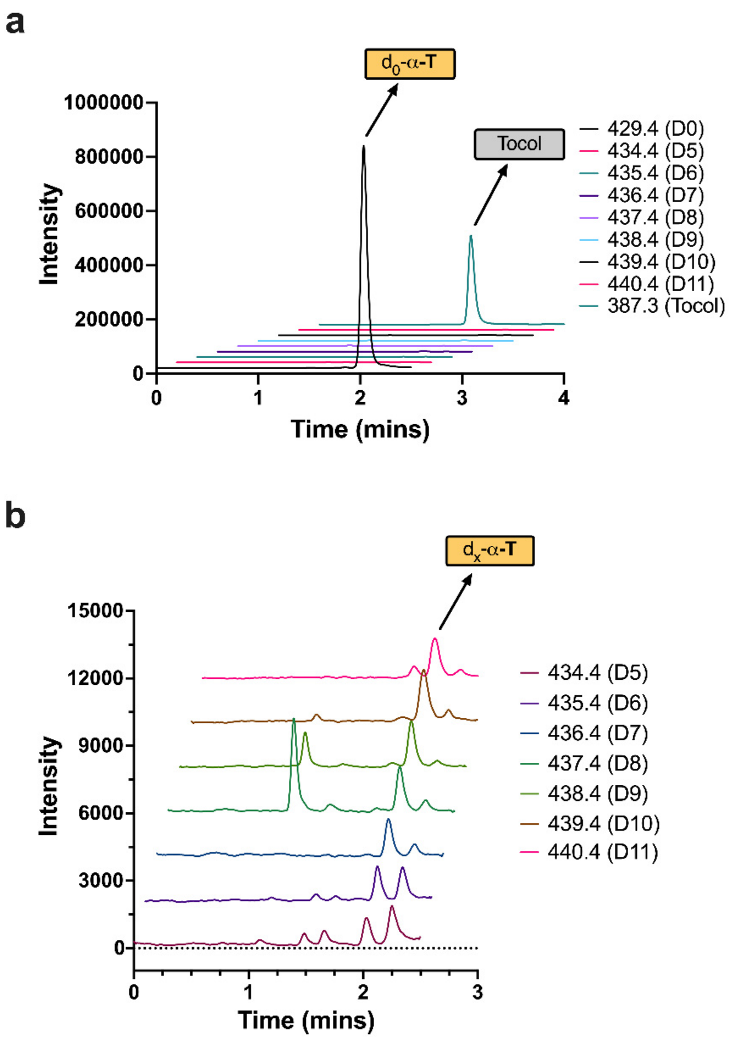

Supplemental Figure S1. Representative LC-MS chromatograms of α-tocopherol (α-T) in plasma collected 12 h after ingestion of intrinsically deuterium-labeled spinach (d_x-spinach) with two eggs. Analyte separation was performed isocratically using methanol as the mobile phase on a C_{18} column. Detection was performed following atmospheric pressure chemical ionization (negative-ion mode). Unlabeled and d_x-α-T were monitored using selected-ion monitoring at mass-to-charge ratios (m/z) 429.4 for d_0-α-T and m/z 434.4-440.4 (1 Da increments) for d_5-d_{11}-α-T. Tocol (internal standard) was monitored at m/z 387.3. (a) Chromatograms showing unlabeled α-T (d_0-α-T), d_5-d_{11}-α-T isotopomers, and the internal standard, tocol. (b) Expanded view of the d_5-d_{11}-α-T chromatograms displayed on a lower-intensity scale for enhanced visualization.

Supplemental Table S1. Food and nutrient composition of controlled diets administered to participants during the 3 days prior to and the initial 12 h of each pharmacokinetic trial.

Diet day	Food item and amount	Meal	Energy (kcal)	Carbohydrate (g)	Protein (g)	Fat (g)	α-T (mg)
Day 1	Croissant, cheese; 55 G	Breakfast	217	21.9	4.6	12.4	0.37
Day 1	Ham, deli; 30 G	Breakfast	44	0.5	6.3	1.7	0.08
Day 1	American cheese, processed; 4 G	Breakfast	15	0.2	0.7	1.2	0.03
Day 1	Quaker Instant Oatmeal Flavored; 1 packet - prepared	Breakfast	160	33.0	4.3	2.3	0.19
Day 1	Buns, wheat; 3 OZ	Lunch	231	39.5	7.3	5.4	0.42
Day 1	Turkey, deli; 4.5 OZ	Lunch	140	2.8	22.2	3.8	0.19
Day 1	American cheese, processed; 19 G	Lunch	70	0.9	3.4	5.8	0.15
Day 1	Carrots, raw; 3 OZ	Lunch	35	8.1	0.8	0.2	0.73
Day 1	Chips - Pringles; 0.74 OZ	Lunch	102	12.9	1.2	5.3	1.46
Day 1	Dressing for salads, ranch; 12 G	Lunch	52	0.7	0.2	5.3	0.27
Day 1	Dole Fruit Bowls Tropical Fruit in 100% Fruit Juice; 2 servings	Snack	148	39.8	0.3	0.4	0.34
Day 1	Snyder's of Hanover Pretzels – Mini; 52 G	Snack	192	40.3	5.5	0.5	0.03
Day 1	Pepperidge Farm Goldfish - Cheddar; 28 G	Snack	133	18.8	3.6	4.6	0.59
Day 1	Banquet Chicken Strips Meal; 1 package - each 8.9 OZ	Dinner	441	44.9	22.9	18.9	1.26
Day 1	Michelina's Authentico Cheeseburger Mac; 1 package - each 8 OZ	Dinner	334	38.7	17.1	11.9	0.36
Day 1	Hawaiian Rolls; 37.75 G	Dinner	122	20.6	3.2	3.1	0.12
Day 1	Butter, salted; 0.17 OZ	Dinner	35	0.0	0.0	3.9	0.11
		**Day 1 Total**	**2468**	**323.7**	**103.7**	**86.7**	**6.70**
Day 2	Pancake, buttermilk, frozen; 3 medium	Breakfast	287	46.4	6.4	8.4	0.76
Day 2	Pancake syrup; 1.4 OZ	Breakfast	102	27.6	0.0	0.0	0.00
Day 2	Sausage, turkey and pork, 54 G	Breakfast	132	0.3	9.7	10.1	0.23
Day 2	Yoplait Original Yogurt - vanilla; 1 container - each 6 OZ	Breakfast	173	32.4	7.4	1.8	0.03
Day 2	Turkey, breast, processed; 4.5 OZ	Lunch	140	2.8	22.2	3.8	0.19
Day 2	American cheese, process; 38 G	Lunch	139	1.8	6.9	11.7	0.30
Day 2	Hellman's & Best Foods Real Mayonnaise; 0.38 FO	Lunch	67	0.1	0.1	7.4	0.32
Day 2	Libby's Juicy Juice - Apple; 4.23 FO	Lunch	60	14.8	0.1	0.2	0.01
Day 2	Buns, wheat; 3 OZ	Lunch	231	39.5	7.3	5.4	0.42
Day 2	Sun Chips - Original; 1 OZ	Lunch	136	18.8	2.3	6.0	1.11
Day 2	Marie Callender's Slow Roasted Beef; 12.3 OZ	Dinner	281	31.0	16.8	10.0	0.43
Day 2	Minute Ready to Serve Brown Rice; 1 cup	Dinner	250	47.8	5.1	4.1	0.51
Day 2	Soup, tomato, cream; 7 OZ	Dinner	113	20.9	2.7	2.6	1.22
Day 2	Nature Valley Crunchy Granola Bar - Oats 'n Honey; 1 bar - each 0.75 OZ	Snack	91	14.4	2.2	3.0	0.42
Day 2	Snack Factory Pretzel Crisps - Original Minis; 1 OZ	Snack	112	23.8	3.0	0.3	0.02
Day 2	Air Heads; 22 G	Snack	86	20.1	0.0	1.0	0.08
Day 2	Peanuts, honey roasted; 1 OZ	Snack	161	6.2	7.2	13.4	1.77
		**Day 2 Total**	**2562**	**348.8**	**99.4**	**89.2**	**7.8**
Day 3	French toast, frozen, regular; 120 G	Breakfast	259	38.4	9.3	7.4	0.73
Day 3	Tennessee Pride Turkey Sausage Patty; 1 serving	Breakfast	111	1.0	12.1	6.5	0.07
Day 3	Pure maple syrup; 1.4 OZ	Breakfast	103	26.6	0.0	0.0	0.00
Day 3	Libby's Juicy Juice Apple; 4.23 FO	Breakfast	60	14.8	0.1	0.2	0.01
Day 3	Lean Cuisine Favorites Macaroni & Cheese; 205 G	Lunch	217	34.6	9.9	4.1	0.12
Day 3	White Castle cheese slider; 104 G	Lunch	283	21.5	13.8	15.4	0.30

Diet day	Food item and amount	Meal	Energy (kcal)	Carbohydrate (g)	Protein (g)	Fat (g)	α-T (mg)
Day 3	Ketchup regular; 18 G	Lunch	18	4.9	0.2	0.0	0.26
Day 3	Carrots raw; 3 OZ	Lunch	35	8.1	0.8	0.2	0.73
Day 3	Yoplait Original Yogurt fruit flavors; 6 OZ	Lunch	173	32.4	7.4	1.8	0.03
Day 3	American cheese, processed; 24 G	Snack	88	1.1	4.4	7.4	0.19
Day 3	Turkey breast processed; 24 G	Snack	26	0.5	4.2	0.7	0.04
Day 3	Crackers, Nabisco Ritz; 19 G	Snack	98	11.8	1.4	5.0	0.73
Day 3	Nabisco Oreo Chocolate Sandwich; 29 G	Snack	137	20.8	1.3	6.0	0.95
Day 3	Gardetto's Snack Mix Original Recipe; 1.75 OZ	Snack	233	32.2	4.4	9.5	0.80
Day 3	Nature Valley Trail Mix Chewy Granola Bar Fruit & Nut; 1.2 OZ	Snack	140	25.0	3.0	4.2	1.08
Day 3	Dole Fruit Bowls Tropical Fruit in 100% Fruit Juice; 1 serving	Dinner	74	19.9	0.1	0.2	0.17
Day 3	Butter salted; 0.17 OZ	Dinner	35	0.0	0.0	3.9	0.11
Day 3	Hawaiian Rolls; 1 each	Dinner	100	16.9	2.6	2.5	0.10
Day 3	Stouffer's Classics Meatloaf; 280 G	Dinner	324	24.8	20.5	16.1	0.60
		**Day 3 Total**	**2514**	**335.4**	**95.7**	**91.1**	**7.03**
Trial Day	Assigned test meal	Breakfast	-	-	-	-	-
Trial Day	Applesauce, sweetened; 4 OZ	Lunch	77	19.8	0.2	0.2	0.2
Trial Day	Banquet Pot Pie Chicken; 7 OZ	Lunch	354	34.0	11.8	18.5	0.4
Trial Day	Banquet Spaghetti & Meatballs; 10 OZ	Lunch	318	31.4	16.7	14.0	1.3
Trial Day	Libby's Juicy Juice Apple; 4.23 FO	Lunch	60	14.8	0.1	0.2	0.0
Trial Day	Goldfish Cheddar; 28 G	Snack	133	18.8	3.6	4.6	0.6
Trial Day	Mozzarella cheese, part skim; 2 sticks	Snack	167	3.2	13.5	11.2	0.3
Trial Day	Pretzel Crisps Original; 3 OZ	Snack	336	71.5	8.9	0.8	0.1
Trial Day	Air Heads; 2 miniature	Snack	88	20.7	0.0	1.0	0.1
Trial Day	Yoplait Light Fat-Free Yogurt fruit flavors; 6 OZ	Dinner	100	19.9	5.2	0.2	0.0
Trial Day	Stouffer's Classics Roast Turkey Breast; 16 OZ	Dinner	440	46.9	26.4	16.0	1.3
Trial Day	Sun Chips Original; 1 OZ	Dinner	136	18.8	2.3	6.0	1.1
Trial Day	Murray Sugar Free Shortbread; 8 each	Dinner	129	21.2	2.0	5.1	0.6
		**Trial Day Total**	**2338**	**321.0**	**90.7**	**77.8**	**5.91**
